# Reducing social media use via contingency management: A replication and extension

**DOI:** 10.1002/jaba.70061

**Published:** 2026-04-07

**Authors:** Dilara Olgun, Meghan A. Deshais, SungWoo Kahng, Robert H. LaRue

**Affiliations:** ^1^ Department of Applied Psychology Graduate School of Applied and Professional Psychology, Rutgers University Piscataway NJ USA

**Keywords:** contingency management, smartphone addiction, social media use

## Abstract

Public health concerns about social media use (SMU) have generated interest in the development of interventions capable of reducing SMU. In this study, we replicated and extended Stinson and Dallery (2023) by evaluating the effects of a contingency management with alternative activity selection intervention on SMU and engagement in alternative activities with four college students. Results from the current study replicate the findings of Stinson and Dallery. Reductions in SMU were observed, but no discernable influence on engagement in alternative activities occurred during the intervention phase. We extended prior work by objectively measuring participant engagement in alternative activities and measuring participant SMU using data collection procedures that potentially improve the accuracy and reliability of SMU measurement.

Limitations of the current study and directions for future research are discussed including the maintenance and generality of contingency management interventions for SMU and considerations for large‐scale implementation.

Smartphones allow users to immediately access a variety of positive reinforcers (e.g., pleasurable activities, communication) with little response effort (O'Donnell & Epstein, 2019). As a result, many individuals engage in heavy levels of smartphone use and, by extension, the applications (apps) on their smartphones. Social media apps, such as Instagram, Facebook, and TikTok, allow users to share ideas, photos, videos, information, or other content electronically (Cambridge Dictionary, n.d.; Encyclopaedia Britannica, n.d.). Social media use (SMU) via smartphone refers to time spent on social networking sites or apps using an individual's smartphone (Stinson & Dallery, 2023). Average daily social media use has increased over time, with global estimates increasing from approximately 111 to 141 min per day from 2015 to 2025 (Statista, 2025). Research indicates that adolescents who spend more than 3 hr per day on social media are more likely to report high levels of internalizing problems such as negative emotions than individuals who spend less time on social media (Riehm et al., 2019). Relatedly, SMU duration is linked to depression and other psychiatric disorders as well as lower academic performance (Andreassen et al., 2012; Junco, 2012; Keles et al., 2020; Riehm et al., 2019; Rosen et al., 2013; Sampasa‐Kanyinga et al., 2019; Shannon et al., 2022). High levels of SMU are also associated with an increased likelihood of sleep disturbances among young adults in the United States (Levenson et al., 2016). Although conclusions about causality cannot be drawn from correlational research, a small body of experimental research indicates that limiting SMU can lead to reductions in depression symptoms (May et al., 2025) and improvements in body image (Thai et al., 2024).

Although there is disagreement among researchers concerning whether heavy SMU should be characterized as an addiction, several nomenclatures have been used to describe substantial daily use of social networking sites including social media addiction (Andreassen et al., 2012; Şahin, 2018), social media disorder (van den Eijnden et al., 2016), social network addiction (Griffiths, 2012), Facebook addiction (Andreassen et al., 2012), internet addiction (Young, 2004), and problematic social media use (Stinson & Dallery, 2023).

Given the concerns about heavy SMU, behavior analysts have begun designing and assessing interventions that are directed at decreasing SMU and smartphone use more broadly (e.g., Williams‐Buttari et al., 2023). For example, Stanley et al. (2022) assessed the effects of a brief contingency management (CM) intervention targeting smartphone use (consisting primarily of SMU) in which participants received online vouchers for meeting use reduction goals. The results indicated that participant smartphone use was lower during the CM condition relative to baseline, suggesting that SMU is amenable to behavioral intervention. In light of the growing public concern about heavy SMU, additional empirical research on behavioral interventions designed to reduce SMU could be valuable.

Prior research suggests that CM, a consequence‐based intervention in which participants earn financial incentives for providing objective evidence of behavior change, can be effective at reducing SMU (Stanley et al., 2022; Stinson & Dallery, 2023). Findings from existing CM research on SMU align with the reinforcer‐pathology model of smartphone use (Hayashi, 2024), which characterizes smartphone use as an impulsive choice that can be modified by reinforcing a self‐control choice. Stinson and Dallery (2023) evaluated the effects of a package intervention that included CM, application limits, and selection of alternative activities on daily SMU duration and engagement in alternative activities. Results indicated that the package intervention produced decreases in SMU duration and decreases in Internet Addiction Test scores for most participants. However, the intervention did not increase participant engagement in their chosen alternative activities. Despite the promising outcomes reported by Stinson and Dallery, their use of a multicomponent intervention package makes it challenging to parse out the isolated effects of each intervention component. Additionally, engagement in participant‐selected alternative activities was measured via self‐report, which is prone to bias and may not accurately reflect actual engagement. To address these limitations, the purpose of the current study was to replicate and extend Stinson and Dallery by (a) evaluating the effects of CM and selection of alternative activities on SMU and (b) objectively measuring participant engagement in alternative activities.

## METHOD

### Participants

Three undergraduate and one graduate student enrolled in a public university participated in the study. A recruitment flyer and accompanying email was sent to university student listservs, and participants were recruited via email. Participants varied in age, gender identity, Hispanic or Latinx ethnicity, and race. P1 did not provide information regarding age, gender identity, ethnicity, or race. P2 was a 24‐year‐old woman who identified with two or more races and did not identify as Hispanic or Latinx. P3 was a 22‐year‐old woman who identified as Asian or Asian American and did not identify as Hispanic or Latinx. P4 was a 24‐year‐old woman who identified as White and reported Puerto Rican ethnicity. Individuals interested in the study were sent a modified Internet Addiction Test (Stinson & Dallery, 2023) via email.

To participate in the study, individuals had to (a) express a desire to decrease their SMU; (b) be willing to send and receive text messages to and from the researcher during the study; (c) own any type of smartphone with screen‐recording capabilities (or a way to record their screen time use), a screen time recorder in the smartphone's regular factory settings (i.e., iOS Screen Time or Android Digital Wellbeing), or videoconferencing technology (Williams‐Buttari et al., 2023); and (d) a score of 31 or higher on a modified Internet Addiction Test (Young & De Abreu, 2011). The Internet Addiction Test is an instrument designed to assess internet addiction. The original instrument was modified for the current study by replacing references to “internet” with “social media.” The 20‐item scale is rated on a 5‐point Likert‐type scale (1 = *never* to 5 = *always*), yielding an overall score between 0 and 100. Scores were categorized as normal use (0–30), mild addiction (31–49), moderate addiction (50–79), or severe addiction (80–100). A cutoff score of 31 was applied, consistent with Young and De Abreu (2011), to identify participants exhibiting at least mild levels of addiction, representing the threshold at which SMU may begin to produce functional impairment. Smartphone ownership rather than ownership of a specific device type served as the inclusion criterion.

### Materials

The study was conducted remotely. The researcher and participants communicated via email, text messages, phone calls, and videoconferencing (Zoom Video Communications). In the current study, SMU was monitored using the iOS Screen Time application because all enrolled participants used iOS devices; had a participant used an Android device, the built‐in Digital Wellbeing application would have been used.

### Dependent variables

The primary dependent variable was *daily SMU*. Daily SMU was the total duration (minutes) per day spent on social media apps on the participant's smartphone (Stinson & Dallery, 2023). Social media apps are apps in which individuals can post or send and receive messages, images, and videos to others, excluding apps used for one‐on‐one messaging (i.e., FaceTime, email, text messages, WhatsApp, or Telegram). To determine daily SMU, the total duration of time spent on each social media app was summed. The secondary dependent variable was *daily enrichment*, which was the total duration (minutes) per day spent on alternative smartphone activities (described below). Daily SMU and daily enrichment data were obtained from screen recordings participants submitted to the researcher via a daily Qualtrics link.

### Interobserver agreement

A secondary observer collected data on daily SMU and daily enrichment during 50% of baseline and intervention days. To train the observers, a simplified version of behavioral skills training (Parsons et al., 2012) was used. First, the researcher provided the observers with a written description of the data collection procedures. Second, the observers practiced collecting data from training videos; 100% correct scoring across two training videos was required to collect data during the study. We used exact agreement to calculate interobserver agreement for daily SMU and daily enrichment. For daily SMU, an agreement was scored when two observers recorded the same total duration within 1 min in either direction to account for rounding and a disagreement was scored when two observers recorded distinct durations (difference greater than 1 min). Interobserver agreement for each variable was calculated by dividing the days with agreement by the total number of days and multiplying the result by 100 to yield a percentage. Agreement was 100% for all participants during baseline and intervention phases.

### Response measurement and observation interval

Daily SMU and daily enrichment were recorded via the Screen Time feature on each participant's smartphone. Screen Time tracks the duration of daily app usage in 24‐hr periods beginning at midnight and ending at 11:59 p.m. Rather than having participants submit photographs (i.e., screenshots) of their smartphone's Screen Time tracker as described by Stinson and Dallery (2023), participants submitted videos (i.e., screen recordings) that displayed the data collected by their Screen Time tracker. They submitted their videos to the researcher in a Qualtrics Survey link provided daily by the researcher via text messages or email. Participants were asked to submit their data each day by 12:30 p.m. For example, data for Tuesday were submitted by 12:30 p.m. Wednesday. To collect prebaseline data, participants provided the researcher archived Screen Time data from the 7 days prior to their intake meeting if their Screen Time feature was turned on during the preceding week.

### Procedural fidelity

Trained observers collected data on the researcher's correct implementation of study procedures during 100% of days. Study steps required that the researcher (a) inform each participant of approaching phase changes between 6:00 p.m. and 8:00 p.m. the evening before the phase began, (b) text each participant a daily Qualtrics link to prompt data submission each day during baseline and intervention phases, (c) prompt participants to upload their submission video if it not received by 1:00 p.m. during baseline and intervention phases, and (d) send each participant a message about their daily earnings and ClinCard balance with visual summary and feedback (i.e., “Congratulations, you have met your daily goal!” or “Unfortunately, you have not met your daily goal.”) each day during the intervention phase. Although feedback was delivered each day, no specific time frame was established for procedural fidelity checks of feedback delivery.

The same procedures used for interobserver agreement were used to train observers to collect procedural fidelity data. Procedural fidelity was calculated by dividing the number of steps scored as “yes” by the total number of steps in each phase and multiplying the outcome by 100 to yield a percentage. When both observers scored the same step as “yes” or “no,” an agreement was scored. When one observer scored a “yes” and the other observer scored a “no” for the same step, a disagreement was scored. Procedural fidelity was 100% for all participants across baseline and intervention phases.

### Technical difficulties

During the study, technical difficulties with video submissions occurred once with P2 on the first intervention day. On that day, P2's Screen Time tracker only displayed usage of the participant's seven most used apps rather than all the apps on their smartphone. The data were retained in the analysis because the recorded SMU still exceeded the participant's daily goal, allowing for a valid measure of goal adherence.

### Experimental design

To replicate Stinson and Dallery (2023), a concurrent multiple‐baseline‐across‐participants design was used to assess the effects of CM on daily SMU and daily enrichment.

### Procedures

#### Intake meeting

Intake meetings were conducted via Zoom videoconferencing with each participant individually. First, the researcher asked the individual if they would like to decrease their SMU. Only individuals that answered in the affirmative were eligible for participation. Second, the consent form was reviewed with an overview and description of each study phase. Third, participants were asked to provide a list of social media apps on their smartphones (e.g., Instagram, Facebook, Tik Tok, Snapchat, X, Be Real, Group Me, LinkedIn, Threads, Tumblr, Pinterest, Starmaker). Fourth, participants were asked to choose three daily enrichment activities that they would like to engage in as alternatives to SMU from an adapted Behavior Allocation Survey (Stinson & Dallery, 2023). The Supporting Information includes the adapted Behavior Allocation Survey, which listed activities that could be objectively measured by the participant's smartphone. Importantly, participants had to select activities or apps that they already had on their smartphone. For example, if a participant selected “using a meditation app” as one of their daily enrichment activities, their daily use of the meditation app was obtained from Screen Time submission videos. Fifth, the researcher described participant data collection, screen recording, and daily submission videos. The researcher prompted the participant to practice creating and sending a submission video that displayed all data collected by the Screen Time tracker on their smartphone from the previous day. Sixth, study rules were reviewed with the participant, which were (a) no one except the participant was allowed to use their smartphone and (b) the Screen Time tracking feature on their smartphone needed to remain “on” during the study. Finally, participants were asked to provide their daily SMU and enrichment data from the previous 7 days, if available (Stinson & Dallery, 2023). These data served as prebaseline data and were compared with baseline data for possible reactivity effects.

#### Baseline

During the baseline phase, participants submitted daily SMU and daily enrichment for a minimum of 7 days. Baseline data were distinct from prebaseline data. Similar to Stinson and Dallery (2023), participants earned $1 daily for submitting their videos. The baseline phase concluded after a minimum of 7 days of data were collected, and daily SMU was stable via visual analysis.

#### Contingency management intervention

Before the start of the CM intervention phase, the researcher held a brief virtual meeting with each participant, replicating the procedures described by Stinson and Dallery (2023). The researcher asked the participant to select a daily SMU goal for themselves. Daily goals were individualized rather than the same for all participants. To inform their decision, the researcher provided participants with their mean daily SMU during the baseline phase and information about the negative effects of SMU from relevant literature. Participants were also asked to choose three daily enrichment activities that they would like to engage in as alternatives to SMU from the adapted Behavior Allocation Survey. The researcher and participant then briefly discussed potential strategies for promoting engagement in alternative activities.

The same financial incentive schedule described by Stinson and Dallery (2023) was employed in the current study. On the first day of the CM intervention phase, participants earned $7 if they met their daily SMU goal. Earnings increased $1 per day for each consecutive day a participant met their goal, up to $9 per day. The escalating schedule was reset if a participant missed their goal or did not submit their data on a CM intervention day. Participants also earned $1 daily for submitting their videos.

#### Social validity

Participants completed a treatment acceptability questionnaire that collected information about participants' opinions of study procedures and outcomes based on the instrument developed by Williams‐Buttari et al. (2023). The questionnaire included six questions with 5‐point Likert‐type scales and one open‐ended question (see Table 2). The reliability and validity of the treatment acceptability questionnaire are not known. A link to the questionnaire was sent to participants via text messages and was completed online in Qualtrics. Median scores for each questionnaire item were calculated.

## RESULTS

**FIGURE 1:**
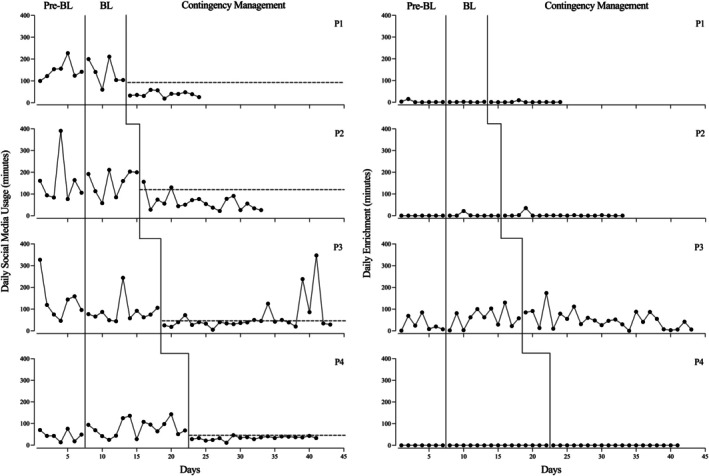
Daily SMU and daily enrichment for participants. The dotted horizontal lines indicate each participant's daily SMU goal.

Figure 1 depicts daily SMU and daily enrichment for all four participants across prebaseline, baseline, and CM intervention phases. Daily SMU during the prebaseline and baseline phases was variable for all participants. During the CM intervention phase, daily SMU decreased for all participants. Daily SMU was at or below participants' SMU goals for most of the CM intervention phase. Overall, participants showed low levels of daily engagement in their selected alternative activities throughout the study, with no notable change from baseline to CM intervention. Table 1 shows the selected alternative activities for all participants.

**TABLE 1:** Daily enrichment activities selected by participants.

ID	Activity 1	Activity 2	Activity 3
P1	Meditation app (Calm)	Music/Podcast app (Spotify)	Puzzle app (NYT Games)
P2	Meditation app (Medito)	Exercise app (Steps)	Podcast app (Podcast)
P3	Reading app (Good Reads, iBook)	Exercise app (Splits Training)	Music app (Tidal)
P4	Education app (Duolingo)	Puzzle app (Logic Puzzles)	Journal app (Journal)

*Note*: ID = Participant identification number.

P1 exhibited high and variable daily SMU during the prebaseline and baseline phases. Following the introduction of the CM intervention, daily SMU decreased, and P1 met their daily goal during each day of the CM intervention phase. P1's daily enrichment remained low and stable during all phases. P2 engaged in high and variable daily SMU during the prebaseline and baseline phases. During the CM intervention, P2's levels of daily SMU decreased, and they met their daily usage goal on 16 out of 18 CM intervention days. P2's daily enrichment was low during all phases, except 1 day during baseline and 1 day during the CM intervention.

P3 exhibited variable levels of daily SMU during the baseline and prebaseline phases. During the CM intervention phase, P3's daily SMU decreased but became increasingly unstable toward the end of the CM intervention phase. They met their daily SMU goal on 20 out of 24 CM intervention days. P3's daily enrichment was variable across all phases. The CM intervention phase was terminated for all participants contingent on consistent levels of SMU at or below daily use goals as determined by visual analysis. Daily SMU for P3 exceeded their daily goals on several occasions during the CM intervention phase, so it was extended to allow for additional data collection.

P4's daily SMU was variable during the prebaseline and baseline phases, with an increasing trend. In the CM intervention phase, P4's daily SMU decreased and remained stable throughout the CM intervention phase. They met their daily SMU goal in 18 out of 19 CM intervention days. Daily enrichment remained at zero throughout all phases. Overall, consistent decreases in daily SMU were observed for all four participants during the CM intervention phase. No change was observed in daily enrichment for any participants.

**FIGURE 2:**
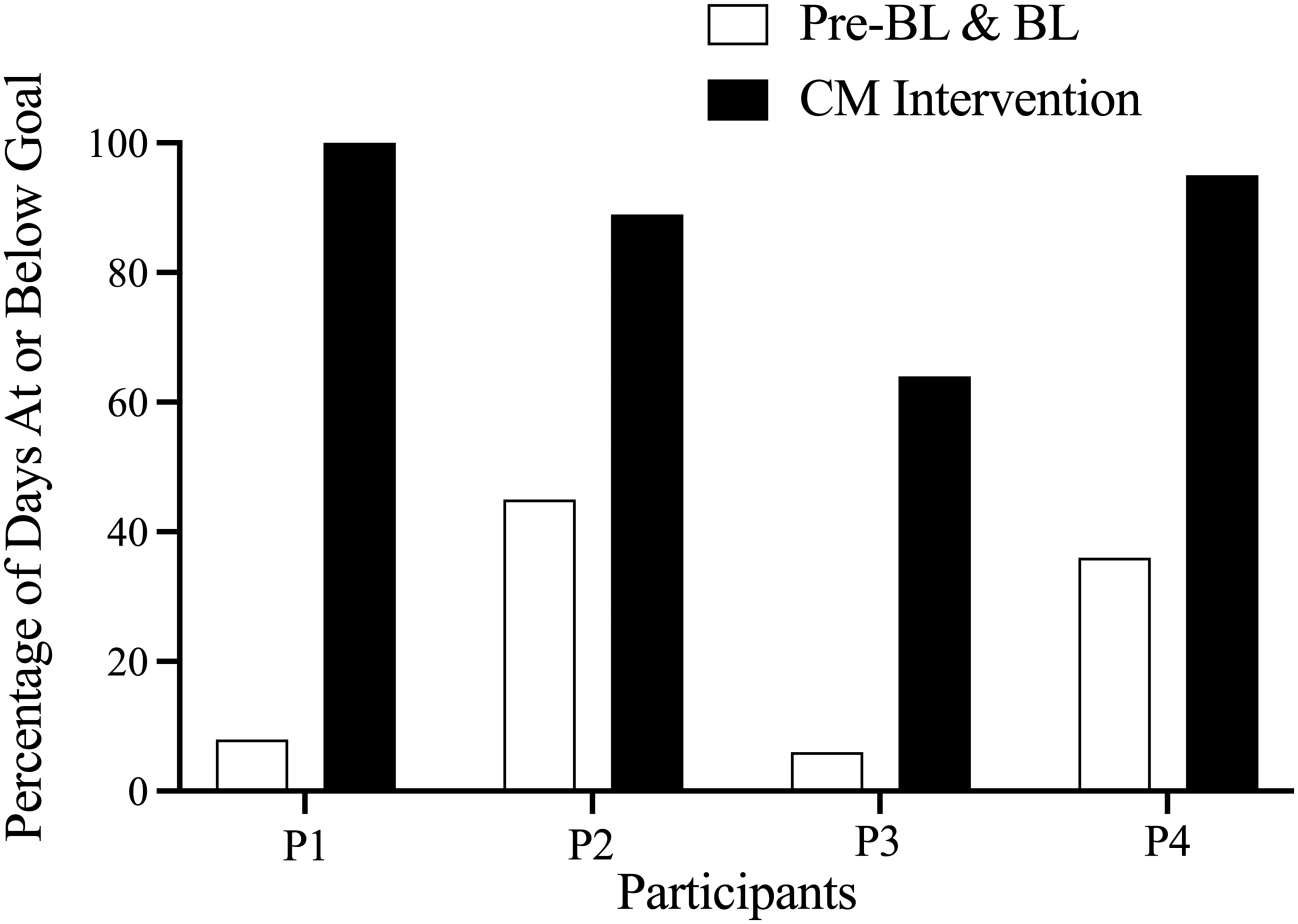
Percentage of days at or below daily SMU goals for participants. PreBL = prebaseline, BL = baseline.

Figure 2 displays the percentage of days at or below each participant's daily SMU goal during baseline and CM intervention phases. Prebaseline and baseline data are combined in this figure. Participant daily SMU goals were 90 min for P1, 120 min for P2, and 45 min for P3 and P4. During the study, P1's daily SMU was at or below their daily goal in 8% of baseline days and 100% of CM intervention days. For P2, daily SMU was at or below their daily goal during 45% of baseline days and 89% of CM intervention days. Daily SMU for P3 was at or below their daily goal during 6% of baseline and 64% of CM intervention days. For P4, daily SMU was at or below their daily goal on 36% of baseline days and 95% of CM intervention days. Total earnings for each participant were as follows: P1 earned $112, P2 earned $179, P3 earned $197, and P4 earned $205.

**FIGURE 3:**
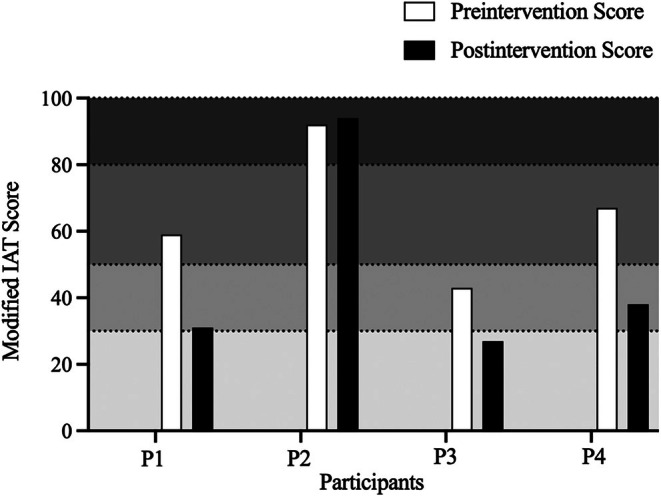
Pre‐ and postintervention modified internet addiction test scores. IAT = Internet Addiction Test. The dotted horizontal lines indicate the scoring categories (0–30: normal use; 31–49: mild addiction; 50–79: moderate addiction; and 80–100: severe addiction), with scores falling in lightest gray reflecting normal use and scores falling in the darker shades indicating mild to severe addiction.

Figure 3 displays the modified Internet Addiction Test scores for participants prior to and following the CM intervention. For three out of four participants, scores on the modified Internet Addiction Test were lower following exposure to the CM intervention. According to the assessment scoring guide, P3's preintervention score met criteria for “mild addiction” and their postintervention score met criteria for “normal use.” P4 and P1's preintervention scores met criteria for “moderate addiction,” and following CM intervention their scores met criteria for “mild addiction.” P2's scores met criteria for “severe addiction” both before and after exposure to the CM intervention.

Table 2 displays treatment acceptability data for the four participants. As noted, the reliability and validity of this measure are not known; therefore, findings concerning social validity and acceptability must be interpreted with caution. Overall, most participants rated the CM and alternative activity selection intervention positively, with most ratings in the 4 to 5 range. Three participants rated the CM intervention very effective, and one participant rated the CM intervention moderately effective at decreasing their SMU. Participants rated the use of smartphone‐based alternative activities as a replacement for SMU moderately reasonable (however, most did not engage in alternative activities). Most of the participants reported being willing to monitor their SMU after the study, and all participants reported that sharing the screen time data with the researcher was not disruptive. In response to the one open‐ended question in the treatment acceptability questionnaire (“Please share any additional thoughts or feelings that you have about participation in this study”), P2 stated, “I found myself opening social media apps a lot but immediately closing them to save my allotted social media time for later, but then would rarely return to it. The study was very effective for me.” P3 responded, it “was difficult at first but, after a few days I adjusted and enjoyed being off my social apps.” Notably, P4 reported,

in my own personal experience, I did use social media significantly less, but I also used my phone significantly less in general. Rather than using alternative apps such as meditation apps or puzzle games, I opted to spend less time on my phone in general and spent more time with my boyfriend, friends, doing school work, and being outside. I do think it was a good idea to encourage the use of alternative apps such as meditation and wellness apps, but I personally didn't use them much.

P1 did not respond to the open‐ended question.

**TABLE 2:** Median treatment acceptability ratings.

Statement	Median score	Range
How acceptable did you find the Contingency Management procedure you experienced during this study?	5	4–5
How effective was contingency management intervention at decreasing your social media use?	5	4–5
How reasonable was the use of smartphone‐based alternative activities as a replacement for social media use?	3	2–4
How acceptable did you find the alternative activities?	4	3–5
How disruptive to your day was sharing your smartphone data with the researcher?*	5	5
How willing are you to monitor your social media use after this study?	4	4–5

*Note*: A score of 5 represented “very acceptable,” “very effective,” “very reasonable,” “very willing,” or “very disruptive.”

* This question was reverse scored.

## DISCUSSION

Research from a variety of disciplines (psychology, psychiatry, public health) indicates that heavy SMU is associated with internalizing problems (Riehm et al., 2019), lower academic performance, poorer sleep, and decreased productivity (Busch & McCarthy, 2021); however, direct casual links have not been established. This study adds to the limited body of empirical research on behavioral interventions designed to decrease heavy SMU. This study also adds to the broader CM literature, providing additional evidence that reinforcement‐based procedures can produce reliable changes in digital behavior.

In this study, we replicated and extended Stinson and Dallery (2023) by evaluating the effects of CM and alternative activity selection on SMU while objectively measuring participant engagement in alternative activities. Stinson and Dallery used a multicomponent intervention package that included CM, notification limits, and selection of alternative activities. Results from the current study replicate the findings of Stinson and Dallery (2023); reductions in SMU were observed, but no discernable influence on alternative activity engagement occurred during the intervention phase. These findings suggest several things. First, the omission of notification limits in the current study suggest that they may not have been an essential component in Stinson and Dallery's intervention package. Second, the lack of change in alternative activities in the current study and Stinson and Dallery suggest that using CM to reduce SMU is not likely to produce concomitant changes in participant engagement in alternative activities. In this study, alternative activity performance was monitored but not directly reinforced. Given that behavior change is often more robust when alternative, desirable responses are systematically reinforced, programming reinforcement for engagement in alternative activities may be necessary to promote change in those responses. Moreover, reinforcing alternative responses in addition to reducing SMU may strengthen overall intervention effects by promoting a more balanced allocation of participant time.

The measurement procedures employed in this study sought to address limitations noted by prior researchers and could inform future work in several ways. First, the data collection procedures used may represent a significant improvement in increasing the feasibility, accuracy, and reliability of SMU measurement. Rather than having participants submit photographs (i.e., screenshots) of their smartphone's Screen Time tracker to the researcher, participants were required to submit videos (i.e., screen recordings). Submission videos entailed participants (a) beginning a screen recording on their smartphone, (b) opening the Screen Time tracker on their smartphone, (c) scrolling from the top of the Screen Time tracker all the way to the bottom, and (d) ending the screen recording. Requiring participants to record the entirety of their Screen Time tracker helped to ensure that usage data from all social media apps on the individual's smartphone were captured. Additionally, when a screen recording is captured on a smartphone, the device owner's name is automatically displayed at the top of the Screen Time Tracker screen, making it more challenging for participants to submit data from another person's smartphone. The use of screen recordings in future studies that are directed at decreasing SMU or smartphone use more broadly may help increase the likelihood of obtaining accurate usage data and decrease the likelihood of data tampering.

Second, participant engagement in their selected alternative activities was objectively measured by participants' smartphones. In prior research, engagement in alternative activities has been measured via participant self‐report (Stinson & Dallery, 2023). Although requiring participants to select alternative activities that can be measured by a smartphone inherently restricts the range of activities to choose from, this approach allows for more precise measurement of engagement. This approach also aligns with a recommendation made by Stanley et al. (2022), who suggested that it would be beneficial to address SMU by encouraging individuals to use apps that promote well‐being rather than social media apps. Notably, two of the four participants in the current study nominated an exercise app as one of their daily enrichment activities. Aside from physical activity apps, most alternative activities that can be measured by a smartphone involve continued use of that smartphone, which may not constitute a meaningful reallocation of participant behavior. Despite the precision gained from objective measurement of alternative activity engagement relative to self‐report, requiring participants to select activities that could be measured by smartphones should be considered a limitation of this study.

Several other limitations of the current study should be noted. First, the financial resources needed to employ the incentive schedule used in this study is a substantial barrier to adoption and implementation. One potential approach to increase the practicality of CM for SMU is to transfer the cost of the intervention to the individuals seeking to reduce their SMU. Deposit contracts in which participants deposit money with the researcher and then earn refunds of their money contingent on meeting behavior change goals capitalize on loss aversion. Deposit contracts have been effective at improving a variety of health‐related responses including problematic smartphone use (Williams‐Buttari et al., 2023), physical activity (Donlin Washington et al., 2016; Krebs & Nyein, 2021; Stedman‐Falls & Dallery, 2020), and smoking (Dallery et al., 2008; Jarvis & Dallery, 2017). There is also some evidence that social incentives (i.e., positive messages from stakeholders) may be a low‐cost, acceptable approach to promoting behavior change (e.g., Ives et al., 2025). It is also possible that gamified versions of CM in which participants earn points or lottery draws (e.g., Petry et al., 2004), try to achieve streak goals, or compete against friends may be effective and more scalable options for treating SMU. The proliferation of commercially available behavior change programs that employ deposit contracts (e.g., QuitBet, DietBet) and gamification provide some evidence of the social validity of these approaches. Future research should explore both the efficacy and uptake of deposit contracts and gamified versions of CM for SMU.

Second, we did not collect follow‐up data to assess the maintenance of behavior change following the end of the CM intervention. A frequent criticism of CM is that intervention effects may not maintain following the discontinuation of financial incentives. A recent meta‐analysis found that CM interventions for substance use produced long‐term improvements in objective measures of substance use up to 1 year after intervention (Ginley et al., 2021), but more research on the long‐term effects of CM on SMU is needed. Preliminary evidence from existing research suggests that treatment effects may not maintain when financial incentives for SMU are removed (Stanley et al., 2022; Stinson & Dallery, 2023). It is possible, however, that intervention duration affected maintenance in prior research. Longer durations of exposure to CM for substance use are associated with improved long‐term outcomes, and the same may be true for CM interventions for SMU. Like substance use, SMU likely provides immediate access to rewarding stimuli, whereas abstaining from SMU or engaging in alternative activities likely does not. Brief exposure to CM for SMU may not provide individuals sufficient exposure to the delayed, natural reinforcers available for reduced SMU and engagement in alternative activities (i.e., better sleep patterns, improved mental or physical health). In addition to collecting follow‐up data to examine maintenance, future researchers could consider incorporating procedures designed to increase the likelihood of maintenance including longer intervention duration and reinforcement thinning during the later stages of intervention.

Third, it is possible that participants accessed social media on other devices (i.e., computers or tablets) during this study. Such usage would not have been captured by the Screen Time tracker in participants' smartphones. We elected not to monitor participants' other devices because Stinson and Dallery (2023) reported that their participants infrequently accessed social media on their computers. Specifically, for six of the seven participants whose computers were monitored, the median value of participant SMU via personal computer was 0 min in each phase. Despite this finding, it may be important for future researchers to consider incorporating additional monitoring methods, such as cross‐device tracking, to obtain more reliable measures of participants' overall SMU.

Results from this replication study provide several potential avenues and considerations for future researchers. Future research should examine the effects of incorporating reinforcement contingencies for alternative activities to determine whether this strategy produces more consistent and robust increases in alternative activity engagement alongside reductions in SMU. Explicitly reinforcing engagement in alternative activities may promote more balanced time allocation and stronger overall intervention effects. Reinforcing engagement in alternative activities may also facilitate contact with naturally occurring reinforcers that could replace the programmed reinforcers (i.e., incentives) for reduced SMU, thereby enhancing maintenance of treatment gains. Second, incorporating device‐based measures or permanent products could improve the accuracy and reliability of both SMU and alternative activity measurement while minimizing opportunities for data tampering. Third, researchers should consider likely implementation barriers, including the financial costs of traditional CM arrangements, participant adherence, and technical challenges associated with monitoring multiple devices. Longer term considerations include exploring scalable and socially valid delivery formats of CM for SMU such as deposit contracts, social incentives, or gamified CM to expand access and support sustained behavior change in larger populations.

Despite the noted limitations, this study extends behavior‐analytic research on CM interventions for SMU. We replicated and extended Stinson and Dallery (2023) by evaluating the effects of CM and alternative activity selection on SMU while objectively measuring participant engagement in alternative activities. Outcomes from the current study align with prior findings that CM has the potential to produce meaningful decreases in SMU. Reductions in SMU occurred without imposing application limits, suggesting that such limits may not be necessary to achieve similar outcomes. However, because the study involved a small sample and a brief intervention period, further research is needed to determine the generality and durability of these effects. Given the growing public health concerns about heavy SMU, additional behavior‐analytic research directed at identifying effective interventions for SMU would be beneficial.

## AUTHOR CONTRIBUTIONS

The first author conceptualized the study, conducted the experiment, analyzed the data, drafted the initial manuscript, and contributed to revisions of the manuscript. The second author contributed to the conceptualization of the study, supervision of the project, data analysis, drafting the initial manuscript, and revisions of the manuscript. The third and fourth authors contributed to conceptualization and methodology.

## ETHICS APPROVAL

This study received approval from the Rutgers University Institutional Review Board and was conducted in accordance with established ethical guidelines for the treatment of human participants.

## CONFLICT OF INTEREST STATEMENT

The authors have no conflicts of interest to declare.

## Data Availability

The data that support the findings of this study are available from the corresponding author upon reasonable request. Supporting Information includes Adapted Behavior Allocation Survey.
